# Antibiotic-Free Expression of γ-Glutamyl Transpeptidase in *Bacillus subtilis* and Process Optimization for L-Theanine Separation and Purification

**DOI:** 10.3390/molecules31091476

**Published:** 2026-04-29

**Authors:** Ran Zhang, Bo Jiang, Ziqun Xiao, Longbei Xiang

**Affiliations:** 1 School of Food Science and Technology, Jiangnan University, Wuxi 214122, China; 2 International Joint Laboratory on Food Safety, Jiangnan University, Wuxi 214122, China

**Keywords:** L-theanine, γ-glutamyl transpeptidase, *Bacillus subtilis*, antibiotic-free expression, separation and purification

## Abstract

L-theanine is a characteristic non-proteinogenic amino acid found in tea leaves and has attracted considerable attention because of its diverse physiological activity and broad application prospects. γ-glutamyl transpeptidase (GGT) can catalyze the synthesis of L-theanine from L-glutamine and ethylamine without ATP consumption, highlighting its advantages for enzymatic production. In this study, a complete process was established for L-theanine production. Through screening of single and dual promoters, the optimal expression combination, PyxiE-PspoVG, was identified. Furthermore, by integrating the *dal* selection marker, an antibiotic-free engineered strain was developed. After flask-level optimization, the GGT activity reached 27.32 U/mL and further increased to 127.37 U/mL in 3 L fed-batch fermentation. Using the fermentation broth as the biocatalyst, fed-batch conversion of 0.6 M L-glutamine and 2 M ethylamine yielded 0.52 M (91.44 g/L) L-theanine within 24 h. Further integration of ceramic membrane filtration, ultrafiltration, nanofiltration, electrodialysis, activated-carbon decolorization and ethanol crystallization afforded a final product purity of 95.6%. This study offers a useful reference for large-scale L-theanine production.

## 1. Introduction

L-theanine, a characteristic non-proteinogenic amino acid in tea leaves, accounts for approximately 1–3% of tea dry weight and is highly soluble in water but poorly soluble in most organic solvents [1]. Extensive studies have demonstrated that it exhibits multiple bioactivities, including anxiolytic, neuroprotective, immunomodulatory, and antitumor effects, as well as the ability to enhance cognition and learning [2,3,4,5,6]. These properties have driven growing interest in its applications in pharmaceuticals, food and nutraceuticals [7]. In addition to its potential as an adjuvant in cancer prevention and treatment, L-theanine has been reported to improve sleep quality in both clinical and healthy populations [8,9]. Its safety profile has also led to its recognition as a generally recognized as safe (GRAS) ingredient by the US Food and Drug Administration (FDA). Consistent with expanding demand, the global L-theanine market was valued at approximately USD 50 million in 2025, and is expected to reach USD 58 million by 2029, representing a compound annual growth rate (CAGR) of 3.7% [10].

Despite its promise, efficient and scalable production of L-theanine remains challenging. Conventional extraction from tea leaves is costly and operationally demanding [11]. Using green tea as the raw material, Chen et al. employed ZJL macroporous ion-exchange resin and obtained L-theanine with a purity of 85.43% and an extraction yield of 0.94% [12]. Zhang et al. first purified green tea extract to 50% L-theanine using a 732 cation-exchange resin column, and further purified L-theanine via preparative HPLC, achieving a yield of 70.4% and a purity of 98% [13]. Chemical synthesis suffers from racemate formation and limited consumer acceptance due to its non-natural origin [14]. Tissue culture offers improved yields over direct extraction, but high cost, slow biomass growth and complex downstream processing continue to impede industrial application [15]. To meet increasing market demand, scalable production of L-theanine is required to ensure a stable supply, with industrial processes placing growing emphasis on product purity and environmental sustainability [16]. Enzymatic synthesis is particularly attractive because it offers high selectivity under mild reaction conditions. Among the candidate enzymes, γ-glutamyl transpeptidase (GGT) is a heterodimeric enzyme that undergoes autocatalytic cleavage to generate a large subunit and a small subunit. It catalyzes the hydrolysis of γ-glutamyl peptide bonds and transfers the γ-glutamyl moiety to amino acids, peptides, or water [17]. GGT exhibits high conversion efficiency in catalyzing the production of L-theanine from L-glutamine and ethylamine, without the need for exogenous ATP or an ATP-regenerating auxiliary enzyme system. Accordingly, it has emerged as a research focus for the enzymatic industrial production of L-theanine [18].

*Bacillus subtilis* is widely regarded as a promising host for food-grade biomanufacturing because of its GRAS status, well-characterized genetic background, high-cell-density fermentation capability, and efficient protein secretion capacity [19,20]. However, for industrial food applications, achieving high-level expression while avoiding antibiotic resistance markers remains a major challenge. Promoter engineering provides an effective means to enhance transcription and secretion of target proteins [21]. Auxotrophic complementation genes provide an effective strategy for the construction of food-grade expression systems without antibiotic resistance markers [22,23]. In this context, the alanine racemase gene is particularly attractive as a selection marker in *B. subtilis*, because D-alanine (*dal*) is essential for peptidoglycan biosynthesis yet is absent from commonly used fermentation media [24,25]. Therefore, a *dal*-based selection system may facilitate the development of an antibiotic-free and food-compatible expression platform. Membrane-based separation technologies, which combine high efficiency with low energy consumption, operational cleanliness and compact modularity, are also well suited to sustainable downstream processing [26].

Against this background, we developed an end-to-end strategy for L-theanine production. This approach integrates upstream enhancement of extracellular GGT expression in *B. subtilis* through promoter engineering and antibiotic-free strain construction with downstream fed-batch synthesis and multistep purification involving membrane separation, electrodialysis, activated-carbon decolorization and ethanol-assisted cooling crystallization. Collectively, this progressive coupling of efficient enzyme supply, intensified synthesis and membrane-based product recovery establishes a practical framework for food-grade L-theanine production.

## 2. Results and Discussion

### 2.1. Promoter Optimization to Enhance GGT Expression

**Figure 1:**
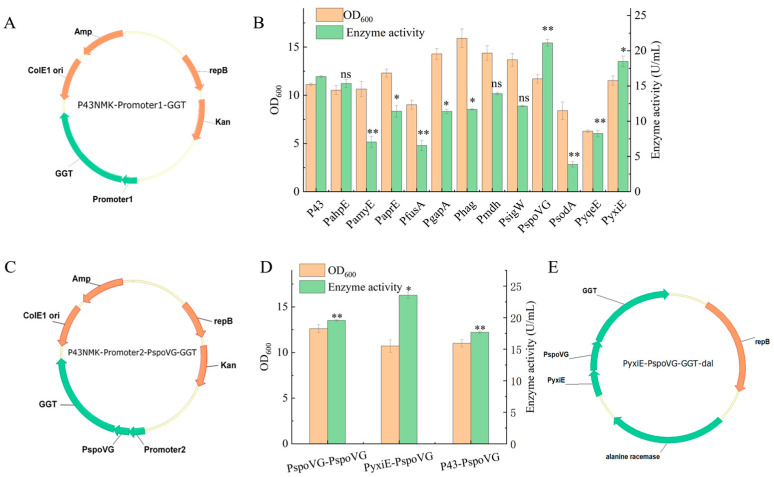
Promoter screening, strain construction and shake flask fermentation. (**A**) Plasmid map of the single-promoter expression strain. (**B**) Enzyme activity and OD_600_ values of recombinant strains harboring different single promoters. The comparison is based on peak extracellular GGT activity during the tested sampling period (12, 24, 36, 48, and 60 h). Data represent mean ± SD of three independent experiments (*n* = 3). Statistical analysis was performed using one-way ANOVA. * *p* < 0.05, ** *p* < 0.01, and ns, not significant vs. the P43 promoter group. (**C**) Plasmid map of the dual-promoter expression strain. (**D**) Enzyme activity and OD_600_ values of recombinant strains harboring different dual-promoter combinations. The comparison is based on peak extracellular GGT activity during the tested sampling period (12, 24, 36, 48, and 60 h). Data represent mean ± SD of three independent experiments (*n* = 3). Statistical analysis was performed using one-way ANOVA. * *p* < 0.05, ** *p* < 0.01 vs. the PspoVG promoter group. (**E**) Plasmid map of the antibiotic-free expression strain.

To further enhance GGT expression in *B. subtilis*, we used our previously constructed recombinant *B. subtilis* strain expressing GGT as the parental strain and screened 12 promoters derived from *B. subtilis* 168 [27]. To reduce production costs and simplify the fermentation process, only constitutive and auto-inducible promoters were considered in this study. The plasmid map of the constructed single-promoter expression strains is shown in Figure 1A, in which Promoter1 denotes one of the 12 different individual promoters. After cultivation in fermentation medium, the OD_600_ of the fermentation broths was measured, and the culture supernatants of the recombinant strains were collected for GGT activity assay and OD_600_ analysis.

As shown in Figure 1B, based on the highest extracellular GGT activity recorded for each recombinant strain across the five sampling time points (12, 24, 36, 48, and 60 h), two promoters showed significantly higher activity than the reference P43 (*p* < 0.05). The GGT activity was measured directly from the culture supernatant. Among them, the recombinant strain carrying PspoVG showed the highest maximum GGT activity (21.11 U/mL), followed by PyxiE (18.49 U/mL), whereas the remaining promoters yielded maximum activities ranging from 3.87 to 15.35 U/mL. These results indicate that, in the same host background, promoter strength is a key determinant of secretory GGT expression, with PspoVG and PyxiE representing the most effective regulatory elements in this system.

Based on the single-promoter screening results, dual-promoter expression systems were constructed by placing PspoVG, PyxiE or P43 upstream of the optimal promoter PspoVG. The plasmid map of the constructed dual-promoter expression strains is shown in Figure 1C, where Promoter2 denotes PspoVG, PyxiE, or P43. After incubation in fermentation medium, the culture supernatants of the recombinant strains were collected for the determination of GGT activity and OD_600_.

The PspoVG–PspoVG combination ranked second (19.56 U/mL), whereas the P43–PspoVG combination showed a lower activity (17.67 U/mL). These findings demonstrate that combinatorial promoter engineering can enhance expression without altering the gene structure. The GGT activities obtained with the dual-promoter constructs PyxiE–PspoVG and PspoVG–PspoVG in this study were higher than the activity reported by Yang et al. (18.65 U/mL), which was achieved through overexpression of the PrsA lipoprotein and enhanced mRNA stability [28].

### 2.2. Construction and Validation of the Antibiotic-Free Expression System

Based on the growth deficiency of D-alanine auxotrophic strains in the absence of exogenous D-alanine, an antibiotic-free expression system using *dal* as the selection marker was constructed [29]. The plasmid map of the constructed antibiotic-free expression strain is shown in Figure 1E. Using the plasmid PyxiE-PspoVG-GGT from the recombinant strain with the highest enzyme activity as the template, the dual-promoter region and the GGT gene were amplified by PCR. The vector backbone was amplified from the laboratory-preserved plasmid pUB-DPE-*dal*. The two fragments were then fused by overlap extension PCR. Before transformation, the constructed *B. subtilis* WB600-*dal*^−^ host strain showed no detectable growth on LB medium without D-alanine supplementation, confirming its dependence on exogenous D-alanine and the stringency of the *dal*-based selection system. The resulting construct was transformed into competent *B. subtilis* WB600-*dal*^−^ cells. Transformants were selected on LB medium without D-alanine, yielding the recombinant strain *B. subtilis* WB600-*dal*^−^/PyxiE-PspoVG-GGT. After cultivation in fermentation medium, the OD_600_ of the fermentation broth was measured, and the culture supernatant of the recombinant strain was collected for the GGT activity assay. The results showed that the enzyme activity reached 20.55 U/mL, while the OD_600_ value was 13.11. The current results support the feasibility of the antibiotic-free system for GGT production, but do not yet allow a strict quantitative conclusion regarding the productivity trade-off relative to the antibiotic-based system. Optimization of the medium composition was carried out to further improve enzyme activity.

### 2.3. Shake-Flask Fermentation Optimization and 3 L Bioreactor Fermentation

**Figure 2:**
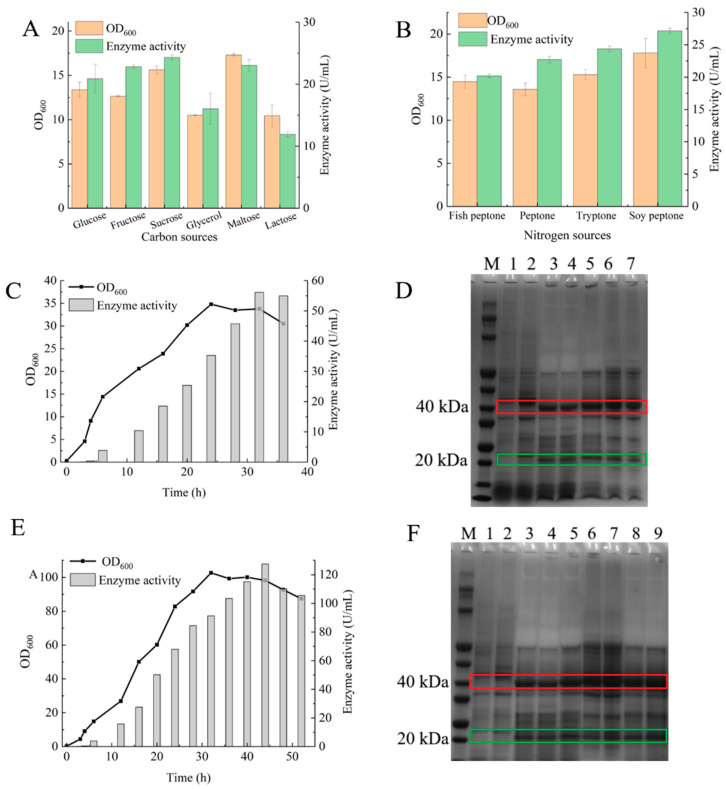
Fermentation optimization and 3 L fermenter cultivation. (**A**) Effects of different carbon sources on the enzyme activity and OD_600_ of the recombinant strain. Data are presented as mean ± SD of three independent experiments (*n* = 3) (**B**) Effects of different nitrogen sources on the enzyme activity and OD_600_ of the recombinant strain. Data are presented as mean ± SD of three independent experiments (*n* = 3) (**C**) Growth curve and enzyme activity during fermentation in a 3 L fermenter (*n* = 1). (**D**) SDS-PAGE analysis of centrifuged fermentation supernatants collected at different time points during fermenter cultivation. Lanes are labeled as follows: M, protein marker; lanes 1–7, fermentation supernatants collected at 3, 6, 16, 24, 28, 32, and 36 h, respectively. The large subunit is demarcated with a red frame, while the small subunit is annotated with a green frame. (**E**) Growth curve and enzyme activity during fed-batch fermentation in a 3 L fermenter (*n* = 1). (**F**) SDS-PAGE analysis of centrifuged supernatants collected at different time points during fed-batch fermentation. Lanes were labeled as follows: M, protein marker; lanes 1–9, fermentation supernatants collected at 3, 6, 16, 28, 32, 36, 44, 48, and 52 h, respectively. The large subunit is demarcated with a red frame, while the small subunit is annotated with a green frame.

To investigate the effects of different carbon sources on fermentation, six carbon sources with equivalent carbon content were compared: glucose (15 g/L), fructose (15 g/L), sucrose (14.2 g/L), glycerol (15.34 g/L), maltose (14.2 g/L) and lactose (14.2 g/L). As shown in Figure 2A, maltose gave the highest OD value, reaching 17.29, whereas sucrose resulted in the highest enzyme activity, reaching 24.33 U/mL. These results indicate that although maltose was more favorable for the growth of the recombinant strain, sucrose was more effective for extracellular GGT production. Therefore, sucrose was selected as the carbon source for subsequent experiments.

To evaluate the effects of different nitrogen sources on fermentation, four commonly used peptones with equivalent nitrogen input conditions were studied. The dosage of each peptone was calculated according to the nitrogen percentage indicated on the product label to ensure that the product of dosage and nitrogen percentage was the same among treatments: fish peptone (12.08 g/L), peptone (10 g/L), tryptone (10 g/L) and soy peptone (16.11 g/L). As shown in Figure 2B, soy peptone yielded both the highest OD value (17.8) and the highest enzyme activity (27.14 U/mL); therefore, it was selected as the peptone source for subsequent fermentation experiments.

Based on the results obtained from shake-flask optimization, the selected conditions were further evaluated in a 3 L bioreactor. In 3 L bioreactor batch fermentation, the pH was maintained at 7.2, the temperature at 37 °C, and the dissolved oxygen level at 30%. The growth curve and extracellular GGT activity of the recombinant strain are shown in Figure 2C. During the first 0–3 h, cell growth was relatively slow and almost no enzyme production was detected. Rapid growth occurred between 3 and 6 h, although enzyme production remained low. From 6 to 24 h, the growth rate decreased, whereas enzyme activity continued to accumulate. Between 24 and 32 h, the culture entered the stationary phase, and the enzyme activity reached a maximum of 56.14 U/mL, which was 2.23-fold higher than that obtained in the optimized shake-flask culture. At 36 h, enzyme activity decreased slightly. Extracellular supernatants collected at 3, 6, 16, 24, 28, 32 and 36 h were further analyzed by SDS–PAGE, as shown in Figure 2D.

In 3 L fed-batch bioreactor fermentation, the pH was maintained at 7.2, the temperature at 37 °C, and the dissolved oxygen level at 30%. A total of 200 mL feeding medium was supplied at a feed rate of 10 mL/h. As shown in Figure 2E, feeding was initiated at 4 h when the OD_600_ reached 9.71, i.e., approximately the preset threshold value of 10. The recombinant strain grew rapidly from 3 to 32 h, accompanied by a continuous increase in enzyme activity. Between 32 h and 40 h, the culture entered the stationary phase. At 44 h, the OD value decreased slightly, whereas the enzyme activity reached a maximum of 127.37 U/mL, which was 5.05-fold higher than that obtained in the optimized shake-flask culture. Thereafter, both enzyme activity and OD declined. Extracellular supernatants collected at 3, 6, 16, 28, 32, 36, 44, 48 and 52 h were analyzed by SDS–PAGE, as shown in Figure 2F.

### 2.4. Fed-Batch Synthesis of L-Theanine

**Figure 3:**
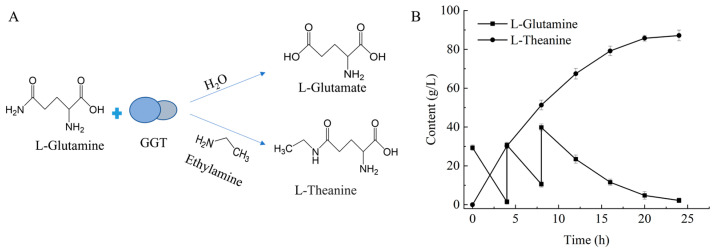
L-theanine synthesis reaction process and fed-batch feeding strategy. (**A**) GGTcatalyzed reaction process for L-theanine synthesis. (**B**) L-theanine synthesis by fed-batch feeding strategy.

The enzymatic synthesis of L-theanine is shown in the reaction process in Figure 3A. To improve substrate conversion efficiency and product formation yield, a fed-batch strategy was adopted to maintain relatively higher substrate ratio (ethylamine: L-glutamine) during the reaction, which is crucial for minimizing the by-product L-glutamate and maximizing L-theanine production. The synthesis was initiated with 0.2 M L-glutamine, 1.6 M ethylamine and 2 U/mL crude enzyme at pH 10.5 and 35 °C. Based on the progress of the reaction, additional L-glutamine and ethylamine (0.2 M each) were supplied at 4 h and again at 8 h. Consequently, the total substrate concentrations reached 0.6 M L-glutamine and 2.0 M ethylamine. To rule out any potential non-enzymatic background formation, enzyme-free and heat-inactivated enzyme controls were included. These controls showed no L-theanine formation, confirming that the observed synthesis was catalyzed by GGT. As shown in Figure 3B, this fed-batch strategy enabled the production of 91.44 g/L (0.52 M) L-theanine within 24 h, corresponding to a yield of 87%. The L-theanine yield achieved in this study exceeds the 0.49 M (85.36 g/L) reported by Shuai et al., and it also surpasses their conversion rate of 66.1% [30].

### 2.5. Optimization of Multistage Membrane Separation for L-Theanine Purification

**Figure 4:**
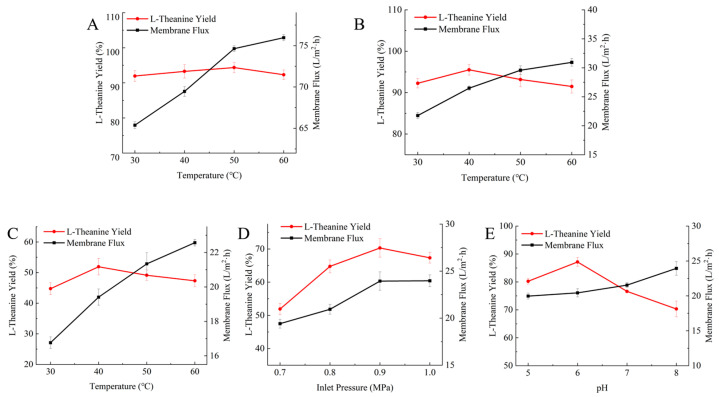
Purification of L-theanine by multistage membrane separation. (**A**) Effect of temperature on L-theanine yield and ceramic membrane flux, (**B**) ultrafiltration membrane flux, and (**C**) nanofiltration membrane flux. (**D**) Effect of transmembrane pressure on L-theanine yield and nanofiltration membrane flux. (**E**) Effect of pH on L-theanine yield and nanofiltration membrane flux.

Ceramic membranes generally have pore sizes in the range of 0.1–10 μm, enabling the effective removal of cell debris, bacteria and other impurities from fermentation broths or hydrolysates. As such, they are often used as a pretreatment step prior to ultrafiltration. Ceramic microfiltration membranes are commonly applied in the pretreatment of extracts or biological suspensions for the recovery of bioactive compounds and to reduce membrane fouling in downstream membrane processes [31]. Temperature was a key factor affecting ceramic membrane performance in this study. As shown in Figure 4A, the membrane flux and L-theanine yield varied with temperature. As the temperature increased, the membrane flux also increased, from approximately 45 L/(m^2^·h) at 40 °C to 75.97 L/(m^2^·h) at 60 °C. However, excessively high temperatures may aggravate membrane fouling through protein denaturation and were accompanied by a slight decline in L-theanine recovery in this study. The highest L-theanine recovery (94.36%) was obtained at 50 °C.

Ultrafiltration is a pressure-driven membrane-based separation process in which porous membranes with pore sizes of 2–100 nm are used to achieve the molecular separation of nanoscale substances [32]. The ultrafiltration membrane was primarily used to retain macromolecular impurities, including proteins and polysaccharides with molecular weights above 3000 Da, thereby further reducing color and turbidity. As shown in Figure 4B, the membrane flux and L-theanine yield of the ultrafiltration membrane varied with temperature. Temperature optimization revealed that 40 °C was the optimal operating temperature, at which the membrane flux reached 30.94 L/(m^2^·h) and the L-theanine recovery was 95.51%. Although increasing temperature improved membrane flux, the recovery declined when the temperature exceeded 50 °C.

The nanofiltration membrane, with a molecular weight cut-off of 300 Da, was mainly used to remove low-molecular-weight organic impurities and part of the dissolved salts. The molecular weight of L-theanine is approximately 174.2 Da. As shown in Figure 4C, the membrane flux and L-theanine yield of the nanofiltration membrane were affected by temperature, with the highest yield (51.91%) obtained at 40 °C. As shown in Figure 4D, both membrane flux and L-theanine yield varied with transmembrane pressure, and the yield increased to 70.31% at 0.9 MPa. As shown in Figure 4E, the membrane flux and L-theanine yield of the nanofiltration membrane also varied with pH, reaching 87.15% at pH 6. Excessively high pressure or pH conditions deviating from near-neutral values both resulted in lower flux and reduced recovery.

### 2.6. Electrodialysis Desalination

Electrodialysis is a membrane separation process in which anions and cations in solution migrate through anion- and cation-exchange membranes, respectively, under a direct current electric field. Owing to the selective permeability of ion-exchange membranes toward ions, electrodialysis can be applied to seawater desalination [33]. The nanofiltration permeate still contained a relatively high salt content, mainly originating from the buffer system and reaction by-products, and electrodialysis was therefore employed for efficient removal of inorganic ions. In the electrodialysis system, the nanofiltration-treated feed solution was introduced into the diluate compartment, 2% NaCl solution was added to the electrode compartment, and water was added to the concentrate compartment. The system was operated under a constant voltage of 30 V. When the conductivity in the concentrate compartment exceeded that in the diluate compartment by tenfold, part of the concentrate was discharged and replaced with an equal volume of Milli-Q water.

**Figure 5:**
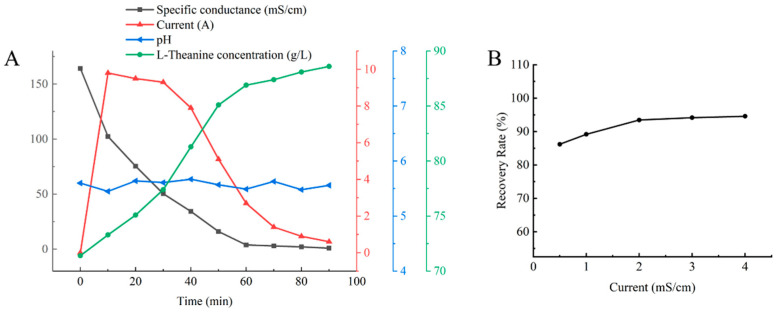
Desalination by electrodialysis. (**A**) Time course of electrodialysis desalination. (**B**) L-theanine yield at different desalination endpoints (*n* = 1).

To monitor the operational behavior of the electrodialysis process during desalination, continuous electrodialysis experiments were carried out using the nanofiltration-treated reaction solution. During the process, the dynamic changes in conductivity, current, pH and L-theanine concentration were systematically examined as functions of time, and the corresponding profiles were plotted. As shown in Figure 5A, the conductivity exhibited a linear decrease over time during the initial stage of desalination. When the conductivity decreased to approximately 4.0 mS/cm, the rate of decline began to slow. A further decrease from 4.0 mS/cm, corresponding to a desalination rate of 97.56%, to approximately 0.4 mS/cm, corresponding to 99.75%, resulted in only limited additional salt removal. Meanwhile, the L-theanine concentration gradually increased during the process. Because the solution volume in the diluate compartment gradually decreased during electrodialysis, the L-theanine recovery could not be accurately determined in real time; therefore, the entire remaining solution was collected at the end of desalination for recovery analysis.

Product recovery after desalination is an important indicator for evaluating electrodialysis performance. To systematically analyze the effect of desalination endpoints, different terminal conductivity values were selected as control variables, namely 0.5, 1.0, 2.0, 3.0 and 4.0 mS/cm. The corresponding L-theanine recoveries are shown in Figure 5B. Conductivity decreased linearly during desalination, and a terminal conductivity of 2.0 mS/cm provided the best balance between product recovery and salt removal, yielding an L-theanine recovery of 93.45% and a desalination rate of 98.78%. Although a lower terminal conductivity (<1.5 mS/cm) resulted in a higher desalination rate, it also led to reduced current efficiency, increased energy consumption and a slight increase in L-theanine loss. Therefore, under the tested conditions, a terminal conductivity of 2.0 mS/cm was selected for subsequent crystallization based on the balance between desalination rate and L-theanine recovery. It should be noted that the terminal conductivity was selected primarily according to electrodialysis performance, and the effect of different desalination endpoints on the purity of the final crystalline product remains to be further investigated.

### 2.7. Activated-Carbon Decolorization and L-Theanine Crystallization

**Figure 6:**
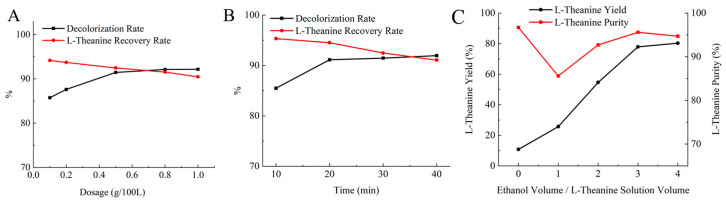
Activated-carbon decolorization and L-theanine crystallization. (**A**) Effect of activated-carbon dosage on decolorization efficiency. (**B**) Effect of decolorization time on decolorization efficiency. (**C**) Effect of ethanol dosage on L-theanine crystallization.

After electrodialysis, the feed solution remained pale yellow, and activated-carbon adsorption was used to remove residual pigments and trace impurities. Several 50 mL aliquots of the feed solution were treated with different dosages of powdered activated carbon and adsorbed at 50 °C for 30 min under constant stirring. After adsorption, the activated-carbon was removed by vacuum filtration, and the filtrate was collected to determine the decolorization rate and L-theanine yield. As shown in Figure 6A, increasing the activated carbon dosage improved the decolorization rate but also led to greater L-theanine loss. At an activated-carbon dosage of 0.5%, the decolorization rate reached 91.46%, while the L-theanine yield remained at 92.47%, indicating a favorable balance between decolorization and product retention.

Adsorption was performed for different decolorization times (10, 20, 30, and 40 min) at an activated-carbon dosage of 0.5% and 50 °C. After decolorization, the activated carbon was removed by vacuum filtration, and the filtrate was collected for analysis. As shown in Figure 6B, the decolorization effect became nearly stable after 20 min. Further prolonging the treatment time did not markedly improve decolorization, but gradually reduced the L-theanine recovery, probably because activated carbon can non-selectively adsorb not only pigments and trace impurities but also a small amount of L-theanine. Considering both the decolorization rate and L-theanine yield, the optimal decolorization time was determined to be 20 min, at which the decolorization rate reached 91.36% and the L-theanine yield was 94.52%.

The solubility of L-theanine in pure water increased only slightly with increasing temperature, making direct cooling crystallization inefficient. Ethanol was therefore used as an antisolvent to reduce solubility substantially; for example, at a water-to-ethanol ratio of 1:3, the solubility of L-theanine at 20 °C was only 3.5 g/100 mL. During crystallization, the temperature was maintained at 60 °C and the stirring speed at 20 rpm. Ethanol was added at 0.5 mL/min, after which the system was cooled to 5 °C at a rate of 5 °C/h and aged for 1 h. The resulting crystals and mother liquor were separated by vacuum filtration, and the recovered crystals were dried, weighed, and analyzed for L-theanine yield and purity. As shown in Figure 6C, without ethanol addition, the process involving only evaporation-induced crystallization and cooling gave a low L-theanine yield of 10.8%. When the ethanol dosage was equal to the volume of the remaining feed solution, the yield increased to 25.7%. As the ethanol dosage increased further, both yield and purity increased. However, when the ethanol dosage reached four times the volume of the L-theanine solution, the purity decreased. When the ethanol dosage was three times the volume of the remaining feed solution, the yield of L-theanine reached 77.9% with a purity of 95.6%, in which L-glutamine and L-glutamic acid accounted for 1.17% and 1.84%, respectively. To clarify the separation efficiency of each step in the L-theanine purification process, the optimal process conditions and corresponding single-step recovery rates for each step were determined in this study. Furthermore, the total recovery rate of L-theanine was calculated to be 54.09% (Table 1).

## 3. Materials and Methods

### 3.1. Strains, Plasmids and Main Reagents

In this study, *Escherichia coli* DH5α was used for plasmid construction, *Bacillus subtilis* WB800 was employed as the expression host for promoter screening, and the genome of *B. subtilis* 168 was used as the DNA template for promoter amplification. To construct an antibiotic-free expression system, the D-alanine auxotrophic strain *B. subtilis* WB600-*dal*^−^ was used. This strain was derived from *B. subtilis* WB600 by deletion of the alanine racemase gene (*dal*). All strains were preserved in our laboratory, and all plasmids used in this study had been previously constructed either in our laboratory or by the authors in earlier work. The primer sequences are listed in Table S1. Primer synthesis and DNA sequencing were performed by Genewiz Biotechnology Co., Ltd. (Suzhou, China). High-fidelity DNA polymerase was purchased from Takara Bio Inc. (Kusatsu, Shiga, Japan). The plasmid extraction kit, FastPure^®^ Gel DNA Extraction Mini Kit, and DNA ligase were purchased from Vazyme Biotech Co., Ltd. (Nanjing, China).

L-γ-glutamyl-*p*-nitroanilide (γ-GpNA), glycylglycine, and ethylamine hydrochloride were purchased from Sangon Biotech Co., Ltd. (Shanghai, China), whereas L-threonine, L-glutamine (L-Gln), and L-glutamic acid (L-Glu) were purchased from Sigma-Aldrich (St. Louis, MO, USA). Activated carbon was purchased from Jiangxi Yuanli Huaiyushan Activated Carbon Co., Ltd. (Shangrao, Jiangxi, China). OPA and OPA buffer were purchased from Thermo Fisher Scientific (Waltham, MA, USA). All other chemicals used in this study were purchased from Sinopharm Chemical Reagent Co., Ltd. (Shanghai, China).

### 3.2. Construction and Screening of Promoter Expression Plasmids

Using the genome of *B. subtilis* 168 as the template, promoter sequences, including PahpE, PamyE, PaprE, PfusA, PgapA, Phag, Pmdh, PsigW, PspoVG, PsodA, PyqeE and PyxiE, were amplified with the primers listed in Supplementary Table S1. The native P43 promoter region in the shuttle vector P43-GGT was removed by PCR-based linearization. The amplified promoter fragments were then ligated into the linearized vector using a one-step cloning kit to generate the expression plasmids.

For promoter screening, a single colony was inoculated into 5 mL of LB medium and cultured at 37 °C with shaking at 220 rpm for 12 h. The seed culture was then inoculated at 2% (*v*/*v*) into 100 mL of fermentation medium supplemented with kanamycin in a 500 mL flask. The fermentation medium consisted of 20 g/L yeast extract, 15 g/L glucose, 10 g/L tryptone, 8 g/L NaCl, 1 g/L MgSO_4_·7H_2_O, and 1 g/L Na_2_HPO_4_·12H_2_O. Cultivation was carried out at 37 °C with shaking at 220 rpm. Samples were collected at 12, 24, 36, 48, and 60 h during fermentation. Optical density was measured at 600 nm, and the supernatants were obtained by centrifugation at 10,000× *g* for 1 min at 4 °C for the GGT activity assay. Samples were collected at 12, 24, 36, 48, and 60 h during fermentation, and extracellular GGT activity was measured at each time point. For each promoter, the highest enzyme activity observed across these sampling time points was defined as the maximum enzyme activity and was used for promoter comparison.

For dual-promoter screening, the plasmid from the strain exhibiting the highest GGT activity was used as the starting plasmid. The plasmid was linearized upstream of the promoter region by PCR, and promoter fragments showing higher activity than P43 were amplified and inserted into the linearized vector using a one-step cloning kit to generate the dual-promoter expression plasmids. The cultivation and screening procedures for the dual-promoter constructs were the same as those used for single-promoter screening.

### 3.3. Construction of an Antibiotic-Free Expression System

Using the plasmid from the dual-promoter recombinant strain with the highest enzyme activity as the template, the dual-promoter region and the GGT coding sequence were amplified by PCR. The vector backbone was amplified by PCR using the laboratory-preserved plasmid pUB-DPE-*dal* as the template [34]. The two amplified fragments were then fused by overlap extension PCR to generate the recombinant DNA construct [35]. The resulting construct was transformed into competent *B. subtilis* WB600-*dal*^−^ cells, and transformants were selected on LB medium without D-alanine, yielding the recombinant strain *B. subtilis* WB600-*dal*^−^/PyxiE-PspoVG-GGT.

### 3.4. Shake-Flask Fermentation Optimization and 3 L Bioreactor Fermentation

The optimization of fermentation conditions was conducted in 500 mL shake flasks containing 100 mL medium, and the 3 L bioreactor fermentation was subsequently performed under the optimized conditions. Under equivalent-carbon-content conditions, the effects of glucose, fructose, sucrose, glycerol, maltose and lactose on the growth and extracellular GGT activity of the antibiotic-free recombinant strain were compared. Under equivalent-nitrogen-content conditions, the effects of commonly used peptones, including fish peptone, peptone, tryptone and soy peptone, on the growth and extracellular GGT activity of the antibiotic-free recombinant strain were evaluated. All shake-flask experiments were performed in triplicate.

Using the optimized fermentation medium, the seed culture was inoculated into a DASGIP 3 L fully automatic bioreactor (Eppendorf Societas Europaea, Hamburg, Germany) containing 1 L of fermentation medium. The agitation speed was set at 50–800 rpm, the aeration rate at 0.5–2 vvm, and the oxygen proportion at 21–100%. The aeration rate, oxygen proportion and agitation speed were automatically adjusted by the bioreactor control system in response to the dissolved oxygen (DO) level to maintain DO at approximately 30%. Samples were collected at different fermentation times to determine cell growth and extracellular GGT activity. Fed-batch fermentation was subsequently initiated when the culture reached an OD_600_ of approximately 10. The feeding solution consisted of 200 mL of fivefold-concentrated fermentation medium, which was supplied at a feeding rate of 10 mL/h. The 3 L bioreactor fermentation was carried out in a single run. Fermentation samples were collected at different time points during 3 L bioreactor cultivation and centrifuged to obtain the supernatants. The supernatant samples were mixed with SDS-PAGE loading buffer, heated, and subjected to SDS-PAGE analysis. Proteins were separated on a 20% polyacrylamide gel and visualized by Coomassie Brilliant Blue staining.

### 3.5. Enzyme Activity Assay

The reaction mixture (2 mL) consisted of 50 μL enzyme solution, 50 mM sodium carbonate buffer (pH 10.5), 30 mM glycylglycine, and 1.25 mM L-γ-glutamyl-*p*-nitroanilide. After incubation at 35 °C for 10 min, the reaction was terminated by adding 200 μL of 1 M HCl. A reaction mixture without glycylglycine but supplemented with an equal volume of water was used as the control. The absorbance of the reaction solution was measured at 410 nm using a spectrophotometer (Mapada Precision 7, Shanghai, China). The amount of released *p*-nitroaniline was calculated from the difference in absorbance between the assay and control groups, and its concentration was determined using a standard curve of *p*-nitroaniline (Figure S1). One unit of enzyme activity (U) was defined as the amount of enzyme required to catalyze the release of 1 μmol of *p*-nitroaniline from γ-GpNA per minute under the assay conditions.

### 3.6. Enzymatic Synthesis of L-Theanine

The 2 L reaction system contained 0.2 M L-glutamine, 1.6 M ethylamine, and 0.2 M carbonate buffer (pH 10.5). After determination of the activity of the recombinant enzyme solution, the culture supernatant containing recombinant enzyme was added to the reaction system to achieve a final activity of 2 U/mL. To confirm that the formation of L-theanine was enzyme-catalyzed, negative controls were included in which the enzyme was either replaced with a buffer solution or heat-inactivated. The reaction was then carried out at 35 °C, with the pH maintained at 10.5 throughout the process. A fed-batch strategy was employed to maintain the substrate concentration at a relatively low level, thereby improving the conversion rate and product yield. Additional L-glutamine and ethylamine were fed at 4 h and 8 h, each at 0.2 M, resulting in final substrate concentrations of 0.6 M L-glutamine and 2.0 M ethylamine. After 24 h of reaction, the L-theanine reaction solution was obtained.

### 3.7. Analysis of L-Theanine Content

Prior to column injection, samples were subjected to pre-column derivatization with OPA. Specifically, 1 μL of sample was mixed with 1 μL of OPA reagent and 1 μL of OPA buffer, followed by a 30 s reaction at room temperature. The derivatized sample was then injected into an Agilent 1260 system (Agilent Technologies, Santa Clara, CA, USA) equipped with a Thermo Scientific Hypersil ODS-2 column (Thermo Fisher Scientific, Waltham, MA, USA). Mobile phase A consisted of 27.6 mM anhydrous sodium acetate (pH 7.2), triethylamine, and tetrahydrofuran mixed at a volume ratio of 1000:0.22:5. Mobile phase B consisted of 175 mM anhydrous sodium acetate buffer (pH 7.2), methanol, and acetonitrile at a volume ratio of 1:2:2. The gradient elution was as follows: 0 min, A/B = 92:8; 17 min, 50:50; 20.1 min, 0:100; and 26 min, returning to 92:8. The column temperature was maintained at 40 °C, the flow rate was 1.0 mL/min, the injection volume was 3 µL, and detection was performed at 338 nm. Quantification was carried out using the external standard method. The retention times of L-glutamine and L-theanine were typically 7.1 min and 10.9 min, respectively.

### 3.8. Separation and Purification of L-Theanine

After completion of the reaction, the enzyme was inactivated by boiling. Residual ethylamine was removed by vacuum evaporation. The pretreated solution was then sequentially filtered through ceramic, ultrafiltration and nanofiltration membranes (Nanjing Jiuanyuan Environmental Protection Technology Co., Ltd., Nanjing, China). The permeate volume per unit time was recorded, and the membrane flux (J) was calculated according to the equation J = V/(S × t), where V is the permeate volume (L), S is the membrane area (m^2^), and t is the time required to collect the permeate (h). The yield of L-theanine was calculated as [(L-theanine content after treatment)/(L-theanine content before treatment)] × 100%.

After nanofiltration, the feed solution was subjected to electrodialysis (Hangzhou Lanran Technology Co., Ltd., Hangzhou, China). The electrodialysis unit consisted of three circulation systems and a membrane stack containing 40 pairs of ion-exchange membranes with an effective membrane size of 200 × 400 mm. The feed solution was added to the diluate compartment, a 2% NaCl solution to the electrode compartment, and water to the concentrate compartment. The flow rates were maintained at approximately 500 L/h for both the diluate and concentrate compartments and 200 L/h for the electrode compartment. Samples were collected at different electrodialysis times. The ion removal rate was calculated as [(conductivity before treatment − conductivity after treatment)/conductivity before treatment] × 100%.

The solution was concentrated under reduced pressure and then decolorized with activated carbon. The OD_440_ values of the solution before and after activated-carbon treatment were measured using a spectrophotometer. OD_440_ was used as an indicator of pigment content, and the decolorization rate was calculated as [(pigment content before decolorization − pigment content after decolorization)/pigment content before decolorization] × 100%. After freeze-drying, the L-theanine sample was reconstituted as a 30% (*w*/*v*) solution. Crystallization was initiated at 60 °C with stirring at 20 rpm, while ethanol was added at a rate of 0.5 mL/min. The temperature was then decreased to 5 °C at a cooling rate of 5 °C/h. After crystallization had stabilized (after 1 h), the crystals were collected by vacuum filtration, dried, weighed, and analyzed for purity and yield. Equal masses of the L-theanine standard and the prepared L-theanine sample were weighed and dissolved to prepare solutions of the same concentration. The purity of L-theanine was calculated as [(L-theanine concentration in the sample)/(L-theanine concentration in the standard)] × 100%.

### 3.9. Data Analysis

The data represent the mean of three replicates, expressed as mean ± standard deviation (*n* = 3), and were analyzed using Origin 2025 (OriginLab Corporation, Northampton, MA, USA), with error bars representing the standard deviation of the three replicates.

## 4. Conclusions

In this study, an integrated process was developed for the enzymatic production of L-theanine, encompassing both upstream GGT production and downstream product purification. Promoter engineering, including single and dual promoter screening, increased extracellular GGT activity in *B. subtilis* to 23.55 U/mL and enabled the construction of an antibiotic-free expression system. Subsequent optimization in shake-flask culture and fed-batch bioreactor fermentation further improved the GGT activity to 127.37 U/mL. A fed-batch synthesis strategy enabled L-theanine production, reaching 91.44 g/L. In addition, a green downstream purification workflow, comprising ceramic membrane filtration, ultrafiltration, nanofiltration, electrodialysis, activated-carbon decolorization and ethanol-assisted cooling crystallization, yielded L-theanine with a purity of 95.6%. These results provide a practical foundation for scalable L-theanine manufacturing. Moreover, although the final crystallized product reached a purity of 95.6%, more detailed characterization of crystal quality and impurity profiles would further strengthen the reliability of the purification results.

## Figures and Tables

**Table 1:** Optimal process conditions and L-theanine recovery rates of each purification step.

Purification Step	Optimal Process Conditions	L-Theanine Recovery Rate
Ceramic membrane filtration	Temperature: 50 °C	94.36%
Ultrafiltration membrane filtration	Temperature: 40 °C	95.51%
Nanofiltration membrane filtration	Temperature: 40 °C; pressure: 0.9 MPa; pH: 6	87.15%
Electrodialysis desalination	Terminal conductivity: 2.0 mS/cm	93.45%
Activated carbon decolorization	Dosage: 0.5% (*w*/*v*); time: 20 min	94.52%
Crystallization	Ethanol volume: 3 times the remaining feed solution	77.9%
Total recovery rate	——	54.09%

## Data Availability

The original contributions presented in this study are included in the article and Supplementary Materials. Further inquiries can be directed to the corresponding authors.

## Supplementary Materials

The following supporting information can be downloaded at: https://www.mdpi.com/article/10.3390/molecules31091476/s1; Table S1: Primers used in this study; Figure S1: Standard curve of p-nitroaniline used for the GGT activity assay.
