# Supplementary material for: Antibiotic-Free Expression of γ-Glutamyl Transpeptidase in Bacillus subtilis and Process Optimization for L-Theanine Separation and Purification
Source: Molecules. 2026 Apr 29;31(9):1476. doi: 10.3390/molecules31091476 (PMC13165169; doi:10.3390/molecules31091476)
Supplement: Supplementary file 1 [file molecules-31-01476-s001.zip › molecules-4256941-supplementary.pdf]

Supplementary Materials

Table S1. Primers used in this study

Primers	Sequence (5'-3')
P43-DF	CTGCAGAAGCTTGGCGTAATCATGGTCATAG
P43-DR	AGTTGCTCAAAAAAATCTCGGTCAGATGTTAC
P_{ahpE}-F	CTTTTTCTTTTTCATTTGACAAAAAATATATATTAATTAATAATTCATA
P_{ahpE}-R	GTGGTGGTGGTGGTGAATGTATATTCCTCCTAAAAATGTATTAGAAAGC
P_{amyE}-F	CTTTTTCTTTTTCATTGATTGTGAAGCTGGCTTACAGAAGAGC
P_{amyE}-R	GGTGGTGGTGGTGGTGTCTTGACACTCCTTATTTGATTTTTTGAAGACT
P_{aprE}-F	CTTTTTCTTTTTCATACGGAAATAGCGAGAGATGATATACCT
P_{aprE}-R	GGTGGTGGTGGTGGTGTCTTTACCCTCTCCTTTTAAAAAAATTCAGAGT
P_{fusA}-F	CTTTTTCTTTTTCATCTGGTGCTGCTGTTAAGAAACGTG
P_{fusA}-R	GTGGTGGTGGTGGTGTGGGTAATTTCTCCTTCCTTATTAGGAAATTG
P_{gapA}-F	CTTTTTCTTTTTCATATCGAGGCTTACTTTAAAAAGCCACG
P_{gapA}-R	GTGGTGGTGGTGGTGGATTGTTTCCTCCTTTAAATAAGTGAGAGATAT
P_{hag}-F	CTTTTTCTTTTTCATGGATTTTTTTATTTTTGTATTAACAAAATCAGAG
P_{hag}-R	GTGGTGGTGGTGGTGTGTTTTGTTCCTCCCTGAATATGTTGTTAAGG
P_{mdh}-F	CTTTTTCTTTTTCATGACTGAAGTGAAATGTTCAGAGTTCG
P_{mdh}-R	GTGGTGGTGGTGGTGGTCTCTCTTCTCCTTTATGGCTAG
P_{sigW}-F	CTTTTTCTTTTTCATTTGGGCTATAGCCAAGCGGTAA
P_{sigW}-R	TGGTGGTGGTGGTGATTTATCTAACCTCTGCCTTCACCG
P_{spoVG}-F	CTTTTTCTTTTTCATACGGACAATATTTTGACACTCACAAACCG
P_{spoVG}-R	GTGGTGGTGGTGGTGAGTAGTTCACCACCTTTTCCC
P_{sodA}-F	CTTTTTCTTTTTCATGAAATGCTGGCGGCAGGTTTAA
P_{sodA}-R	GTGGTGGTGGTGGTGGATAATTCCTCCTTAGTATATATGTACTGAAATG
P_{yqeE}-F	CTTTTTCTTTTTCATAACGAATCGCTTGAAGATGCTC
P_{yqeE}-R	GTGGTGGTGGTGGTGAAATCCACCCTCTTTTAGAATTTGCG
P_{yxiE}-F	CTTTTTCTTTTTCATTGCGTGATCATTTAATTGAAGCGCGCGAAG
P_{yxiE}-R	GTGGTGGTGGTGGTGGCTCTTCCCGCCTTTC

P43-SF	ACGGACAATATTTTGACACTCACAAACC
P_{spoVG}-FF	ATTTTTTTGAGCAACTACGGACAATATTTTGACACTCACAAACC
P_{spoVG}-RR	CAAAATATTGTCCGTAGTAGTTCACCACCTTTTCCCTATATAAAAGC
P_{yxiE}-FF	ATTTTTTTGAGCAACTTGCGTGATCATTTAATTGAAGCGCGCGA
P_{yxiE}-RR	CAAAATATTGTCCGTGCTCTTCCCGCCTTTCGGAC
P43-FF	ATTTTTTTGAGCAACTGGATCCTGATAGGTGGTATGTTTTCGCTTG
P43-RR	CAAAATATTGTCCGTGTACCGCTATCACTTTATATTTTACATAATCGCG
PP-FF	GAGTGATCTTCTCAAAAAATACTACCTGTCTGCGTGATCATTTAATTGAAGCG
PP-RR	CTTTTAAGCCGTCTGTACGTTCCTTAAGTTTTTCGCATTTTTTCAAGTTTACGC CGATT
ZT-FF	GCAATCGGCGTAAACTTGAAAAAATGCGAAAAACTTAAGGAACGTACAGAC GGCTTAAAAG
ZT-RR	CGCTTCAATTAAATGATCACGCAGACAGGTAGTATTTTTTGAGAAGATCACTC

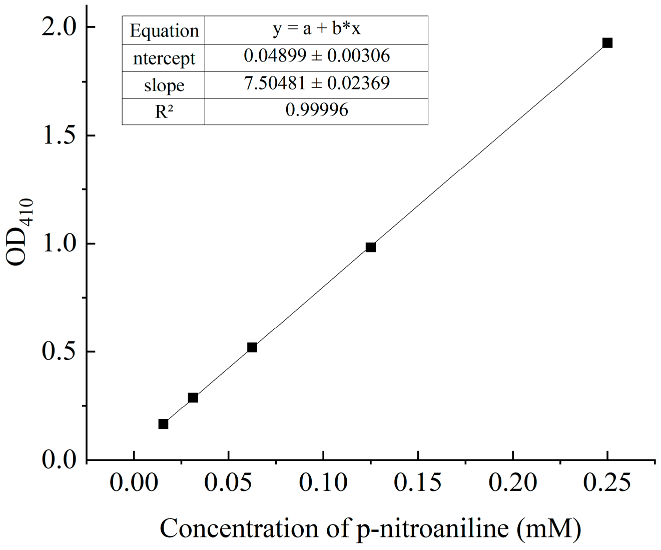

Figure S1. Standard curve of p-nitroaniline used for GGT activity assay.
